# Enumeration and Alertness in Developmental Dyscalculia

**DOI:** 10.5334/joc.55

**Published:** 2019-02-04

**Authors:** Yarden Gliksman, Avishai Henik

**Affiliations:** 1 Department of Psychology and the Zlotowski Center for Neuroscience, Ben-Gurion University of the Negev, Beer-Sheva, IL

**Keywords:** Attention, Numerical cognition, Spatial cognition

## Abstract

Enumeration, the ability to report an amount of elements, differs as a function of range. Subitizing (quantities 1–4) is an accurate and quick process with reaction times (RTs) minimally affected by the number of presented elements within its range. In the counting range (range of 5–9 elements), RTs increase linearly. Subitizing was considered to be a pre-attentive process for many years. However, recently we found that subitizing could be facilitated by improving engagement of attention. Specifically, brief alerting cues increase attentional engagement and reduced RTs in the subitizing range. Moreover, previous studies found that students with developmental dyscalculia (DD) have a smaller than normal subitizing range (3 vs. 4) and their alerting attentional system is impaired. In the current study, we explored whether an alerting cue would increase the subitizing range of adults suffering from DD from 3 to 4. For controls, alerting increased accuracy rates and facilitated enumeration of quantities only in the subitizing range. Participants with DD presented a larger alerting effect; an alerting cue enhanced their RTs in all ranges, but did not increase their smaller than normal subitizing range or accuracy. Our results suggest that both domain-general and domain-specific abilities contribute to the mechanism of enumeration and related to developmental dyscalculia.

## Introduction

In everyday life, numerical information plays a fundamental role in many daily activities (such as calculating change, estimating time) and has an impact on educational and employment achievements (Gevers, Cohen Kadosh, & Gebuis, 2016; Reyna, Nelson, Han, & Dieckmann, 2009). While most people acquire numerical skills in line with their age and developmental stage, about 6% of the population does not (Shalev & Gross-Tsur, 2001). This phenomenon is known as developmental dyscalculia (DD). DD is a learning disability characterized by poor capacity to acquire and use arithmetic skills (American Psychiatric Association, 2013). Children and adults with DD were found to have difficulties in retrieval of arithmetical facts, solving arithmetic equations, using arithmetic procedures (Geary, 2004), and comparing magnitudes (Gliksman & Henik, 2018; Rubinsten & Henik, 2005).

One of the debates regarding learning disabilities, and specifically DD, is whether these disorders involve domain-specific or domain-general deficits. Namely, whether the presented difficulties are related to a specific deficit in numerical abilities, or to deficits in general cognitive abilities, such as language, memory, and attention. Typically, DD was considered to be a unique deficit, and related to a deficit in the intraparietal sulcus (IPS) (Butterworth, 2005, 2010; Dehaene, 1997). Multiple sources of evidence support this assumption. Behavioral studies reported impaired developmental of the “number-sense” in children with DD (Piazza et al., 2010), and imaging studies found that a focal brain injury in the IPS can cause primary acalculia (Ashkenazi, Henik, Ifergane, & Shelef, 2008; Cohen et al., 2018; Gliksman, Naparstek, Ifergane, & Henik, 2017). Those with DD present reduced activity in the IPS when requested to decide which presented array has more elements (Price, Holloway, Räsänen, Vesterinen, & Ansari, 2007), and their IPS was found to be atypical in parameters of length, depth and reduced gray matter volume (Molko et al., 2003; Rotzer et al., 2008). Moreover, a deficient size congruity effect, similar to that shown by those with DD, can be induced by TMS (transcranial magnetic stimulation) to the right IPS (Cohen Kadosh et al., 2007). Additionally, DD can appear without other learning disabilities, and there is evidence that other learning disabilities (e.g., dyslexia) have different cognitive profiles (Cipolotti, 1995; Landerl, Fussenegger, Moll, & Willburger, 2009). Other research provides behavioral and imaging evidence supporting that children and adults with DD present other deficits beside numerical deficits, and that low performance in numerical tasks can be explained by deficits in cognitive mechanisms, such as low memory abilities (Siegel & Ryan, 1989), proactive-interference (De Visscher & Noël, 2013), visuo-spatial working memory (Ashkenazi, Rosenberg-Lee, Metcalfe, Swigart, & Menon, 2013; Geary, Hoard, Byrd-Craven, Nugent, & Numtee, 2007; Rotzer et al., 2009) executive abilities (Bull & Scerif, 2001) and attention abilities (Ashkenazi & Henik, 2010, 2012). Ashkenazi and Henik (2010) examined attention abilities in adults with DD using the attention networks test – interactions (ANT-I) (Callejas, Lupiánez, & Tudela, 2004). Compared to controls, participants with DD presented a larger congruity effect and a larger alerting effect (i.e., impairment in the alerting attentional system, which enables recruiting and maintaining attention). Those results emphasize that deficits in general abilities such as attention may be involved in DD. Importantly, the IPS and other parietal regions were found to be involved in attentional tasks (Corbetta, Patel, & Shulman, 2008; Corbetta & Shulman, 2002; Lau, Rogers, Haggard, & Passingham, 2004; Mevorach, Humphreys, & Shalev, 2006; Mevorach, Shalev, Allen, & Humphreys, 2009; Moos, Vossel, Weidner, Sparing, & Fink, 2012).

Another aspect of the domain-specific vs. domain-general discussion is whether numerical operations are solely related to numerical abilities, or may be influenced by general cognitive processes such as attention or language. For example, it was suggested that familiar arithmetic fact retrieval, such as use of the multiplication table, is related to a general language retrieval mechanism (Dehaene & Cohen, 1995). A similar question can be asked about the operation of enumeration. Enumeration is the ability to report how many items are presented. Enumeration is typically divided into two ranges. When up to 4 items are presented, reaction time (RT) rises slowly, between 40 ms to 100 ms per item (i.e., the RTs slope between the quantities is around zero). It seems that 4 items can be grasped almost simultaneously with no effort. This is termed subitizing (Kaufman, Lord, Reese, & Volkmann, 1949). In contrast, when more than 4 items are presented, RT rises steeply at a rate of 250 ms to 350 ms per item (Jevons, 1871; Trick & Pylyshyn, 1994), and participants are engaged in an effortful counting process. Accordingly, the range of five to nine items is termed the counting range.

One explanation for the subitizing phenomenon is pattern recognition. Arrays of items in the subitizing range create familiar shapes: 2 items create a line; 3 items create a triangle; and 4 items create a square (Ashkenazi, Mark-Zigdon, & Henik, 2013; Mandler & Shebo, 1982). Pattern recognition can be considered a global processing mechanism. Interestingly, global processing of the items that create a familiar shape cannot be applied to larger arrays of objects, or at least not as often as in the subitizing range (Gliksman, Weinbach, & Henik, 2016). Another explanation emphasizes the relation between visuo-spatial working memory and subitizing (Feigenson, Dehaene, & Spelke, 2004; Piazza, Fumarola, Chinello, & Melcher; 2011), and suggests that the origin of the limited capacity is common to subitizing and visuo-spatial memory. Others attributed the distinction between the ranges to attentional resources. It was suggested that subitizing is a preattentive process whereas counting requires attention. Studies demonstrated that subitizing could be performed in parallel with other processes (Atkinson, Campbell, & Francis, 1976; Trick & Pylyshyn, 1994). However, more recent studies found that subitizing is impaired when attentional engagement in the enumeration task is reduced (Egeth, Leonard, & Palomares, 2008; Olivers & Watson, 2008, Piazza et al., 2011).

Participants with DD were found to have a smaller than normal subitizing range (Ashkenazi, Mark-Zigdon, et al., 2013; Schleifer & Landerl, 2011). Namely, the slope of RT changed its trend after 3 items rather than after 4 items. It is not clear whether the source of the smaller than normal subitizing range of those with DD is related to a deficit in the enumeration process or in attentional processes. Ashkenazi and Henik (2012) examined the influence of attentional training on enumeration abilities and on attentional abilities. Training modulated the atypical pattern of DD participants in the attentional test and improved their overall RTs but did not change their smaller than normal subitizing range. Those results support the domain-specific approach, at least with regard to enumeration. Note, however, that this attentional training was designed to improve attentional abilities in general and did not focus on the alerting system specifically.

Recently, we examined whether engagement of the alerting system would enhance the subitizing process (Gliksman et al., 2016). The alerting system of attention regulates the intensity of allocation of attention to a given stimulus (Petersen & Posner, 2012; Posner & Petersen, 1990). Moreover, an alerting cue was found to enhance global processing (Weinbach & Henik, 2011), and a short training procedure aimed to increase alertness level induced a bias towards global processing of attention and reduced local processing (Van Vleet, Hoang-duc, DeGutis, & Robertson, 2011). In our study, participants were presented with an array of dots in the subitizing or counting ranges and were instructed to report the quantity of dots. An alerting cue was presented prior to the presentation of the array in half of the trials. Results indicated that an alerting cue enhances responding only in the subitizing range. The results attributed the benefit of alerting in the subitizing range to enhancement of global processing and pattern recognition (Gliksman et al., 2016).

Taking together that alerting cues were found to enhance attentional bias toward global processing in general (Van Vleet et al., 2011; Weinbach & Henik, 2011) and subitizing in particular (Gliksman et al., 2016), and that those with DD have a smaller than normal subitizing range and deficit in alerting attention, the goal of the current study was to explore whether the impaired subitizing mechanism in those with DD could be improved by engagement of alerting of attention; namely, whether an alerting cue would increase the subitizing range for those with DD. Moreover, because the increase in the subitizing range might be influenced by both domain-specific (enumeration) and domain-general (alerting of attention) abilities, our experiment could shed light on the contribution of those aspects on performance. We used the task reported by Gliksman et al. (2016). Regarding trials without an alerting cue, following previous works (Ashkenazi, Mark Zigdon, et al., 2013; Schleifer & Landerl, 2011), we hypothesized that the DD group would present a smaller than normal subitizing range, resulting in lower accuracy rates and a steeper slope between quantities 3 and 4. Regarding trials with an alerting cue, following previous work (Gliksman et al., 2016), we hypothesized that controls would present an alerting effect only in the subitizing range and not in the counting range. Moreover, we hypothesized that alerting would increase their accuracy rates. For DD participants, following previous works (Ashkenazi & Henik, 2010, 2012), we hypothesized that the DD group would present an increased alerting effect, and that the alerting effect might also be found in the counting range. Regarding the increase of the subitizing range for the DD group, we hypothesized that the alerting cue would increase their smaller than normal subitizing range into 4. Namely, we expected that the trend of the slope would change between 4 to 5 when an alerting cue was presented.

## Method

### Participants

Thirty-four undergraduate students (28 females, mean age 26.7 years old) from Ben-Gurion University of the Negev and Achva Academic College participated in the experiment. Participants were paid in return for their participation (about $8 per hour). All were native Hebrew speakers, with normal or corrected-to-normal vision. All participants gave their informed consent prior to their participation in the study. This study was carried out following the guidelines of the protocol approved by the university’s Ethics Committee. There were two groups of participants with 17 participants in each group: students with DD (14 females, mean age 26.8 years old) and matched control students (14 females, mean age 26.3 years old). The participants in the control group had no reported learning disability. The recruitment of the DD participants was according to the MATAL (Ben-Simon, Beyth-Marom, Inbar-Weiss, & Cohen, 2008). MATAL is a standardized battery built for the diagnosis of learning disabilities (dyslexia, dysgraphia, dyscalculia, and ADHD) in adults (18–35 years old). For recruiting DD participants, 11 relevant computerized subtests were used. For diagnosis of dyscalculia, three subtests were used: computational automaticity (retrieval of simple arithmetic facts), procedural knowledge (mastery of basic arithmetic procedures) and number sense representation (estimated location of numbers on a line). The other subtests evaluated attention and reading abilities. The cut-off criteria for the DD group was 2.5 standard deviations (*SD*) below the average of relevant tests, and average or above in the other tests of attention and reading abilities. The results in the MATAL diagnosis of the DD participants are presented in Table 1 in the Supplementary Materials.

**Table 1:** Percentages of errors for different quantities and groups.

Quantity	Group	

	Control	Developmental Dyscalculia

1	3%	2%
2	2%	2%
3	2%	6%
4	2%	5%
5	10%	19%
6	27%	38%
7	39%	50%
8	43%	51%
9	61%	61%

### Matching controls to DD participants – a preliminary cognitive assessment

The MATAL battery is designed for students suspected of having learning disabilities and is used in higher education institutions. Therefore the control group could not undergo this battery. In order to ensure that control participants did not have any learning disabilities, and to be able to compare the two groups, all participants performed several tests prior to the task, to assess: intelligence (Raven’s Progressive Matrices; Raven, Raven & Court, 1998), reading abilities (Alef-Tav battery; Shany, Lahman, Shalem, Bahat, & Zeiger, 2006) attention abilities (Continuous Performance Test, CPT), and arithmetic abilities (Gliksman et al., 2017). Matching was according to gender and the Raven score. None of the control participants showed any diagnosis of learning disabilities. The description and results of the additional tests are presented in Tables 2–4 in the Supplementary Materials.

### Apparatus

The experiment was run on an IBM-PC with a 17-inch color screen. A headphone set was used to deliver an alerting cue. A microphone was used to register vocal input. RT was recorded electronically by a response box controlled by E-Prime software and was measured from onset of the stimulus to onset of the vocal response. The content of the subject’s vocal response in each trial was input into the computer by the experimenter’s key-press.

### Stimuli

The number of dots varied from 1 to 9 per array. The dot arrays (white on a black background) were created with MATLAB™ code created by Gebuis and Reynvoet (2011). The dots varied in their size and arrangement in each array.

**Figure 1:**
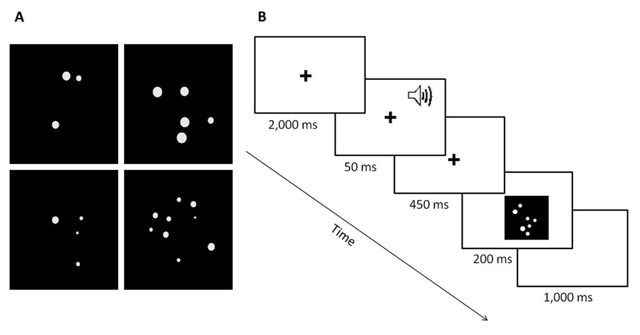
**A)** Examples of stimuli in the experiment. Left column – subitizing range; right column – counting range. **B)** Example of a typical trial.

By controlling those continuous properties, participants could not extract the quantity of the dots from properties such as density, size of the dots, etc. (for further details and reviews see Leibovich & Henik, 2013, 2014; Leibovich, Katzin, Harel, & Henik, 2017). Examples of the stimuli can be seen in Figure 1A. From a viewing distance of 60 cm, the stimulus subtended a visual angle of 8.5°. Each quantity of dots had 18 different arrays. Each array was presented with or without an alerting cue before it appeared.

### Procedure

Participants were instructed to say aloud the number of dots that were presented on the screen, as fast and accurately as possible. Participants did not know what the maximum quantity of dots that could appear would be. A fixation point (a plus sign) was presented for 2,500 ms before the target appeared. In half of the trials, a 50 ms auditory tone was heard through headphones 500 ms before the target appeared. The target replaced fixation and was presented for 200 ms. After a vocal response, the experimenter recorded the number that was said and then a blank screen appeared for 1,000 ms until the fixation point of the next trial appeared (see Figure 1B).

### Design

Each block was composed of 54 trials: 2 (alerting cue) × 9 (quantity of dots) × 3 (repetitions of each quantity). The alerting cue was a 2,000 Hz, 50 ms beep sound. The quantity of dots had nine levels, 1–9, divided into two ranges: the subitizing range (1–4) and the counting range (5–9). A practice block of 9 trials, selected randomly from the full set of trials, was carried out. The practice block was followed by 9 blocks with the trials order randomized. Overall, each participant carried out 486 experimental trials.

## Results

In order to explore the effect of alerting on the subitizing range, three analyses were performed: accuracy rates, RTs and subitizing range.

### Accuracy analysis

Errors were relatively rare in the subitizing range, and increased with the quantity of dots. The percentages of errors for each group are presented in Table 1.

As can be seen, the error rates for the control group in the subitizing range are very low, but this is not the case in the DD group. Namely, those with DD “paid a cost” for larger quantities, even in the subitizing range. To further address the effect of alerting in the different groups and with different quantities, a 2 (alertness) × 8 (quantities) × 2 (group) analysis of variance (ANOVA) was carried out with accuracy rates as a dependent variable. Note, due to the very high error rate in both groups for quantity 9, and specifically, less than 6 correct trials for 7 participants with DD, the quantity of 9 dots was not analyzed. The analyses revealed: a main effect for quantity, as accuracy rates were high and quite similar for quantities 1–4 and decreased with the increase of quantity, *F*(7, 224) = 109.4, *p* < .01, *η_p_^2^* = .77; a main effect for alertness, as accuracies were higher in trials after alerting cues, *F*(1, 32) = 4.3, *p* < .01, *η_p_^2^* = .12; and a marginal effect for group, as controls were more accurate than DD participants, *F*(1, 32) = 3.4, *p* = .07, *η_p_^2^* = .1. The interaction between quantity and group was marginally significant, *F*(7, 224) = 2, *p* = .06, *η_p_^2^* = .05. A planned comparison, separately for each quantity, revealed that controls were more accurate for the quantities 4 and 5, and had a marginal significant effect for the quantity of 3 (3: *F*(1, 32) = 2.5, *p* = .1, *η_p_^2^* = .07; 4: *F*(1, 32) = 6.3, *p* = .02, *η_p_^2^* = .16; 5: *F*(1, 32) = 5.7, *p* = .02, *η_p_^2^* = .15). The interaction between alertness and group was not significant, *F*(1, 32) = 2, *p* = .17, *η_p_^2^* = .05. A planned comparison of the effect of alertness separately for each group revealed that there was a general benefit in accuracy when an alerting cue preceded the target only in the control group: controls – *F*(1, 32) = 6, *p* = .02, *η_p_^2^* = .15; DD – *F* < 1. The interactions between alerting and quantity, and alerting, quantity and group were not significant, *F* < 1. To summarize, participants with DD had lower accuracy rate, in particular for 3, 4 and 5 dots, and alerting cues did not improve their performance.

### RTs analysis

**Figure 2:**
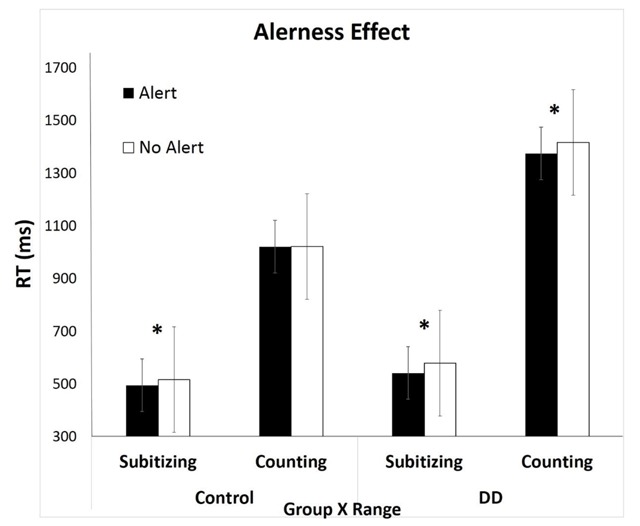
The alerting effect as a function of group and range. Asterisk (*) marks a significant difference. The alerting effect appears in the subitizing range for both groups (i.e., the difference between the black and the white bars), but in the counting range, there is an alerting effect only for the DD group, and not for the controls.

The following analyses were carried out for correct responses only. For each participant in each condition, RTs that were 2.5 *SD* below or above average were excluded. The subitizing range included quantities of 1–4 dots, and the counting range included quantities of 5–8 dots. In order to examine the alerting effect on RTs in different groups and for different ranges of quantities a 2 (alertness) × 2 (range) × 2 (group) ANOVA was carried out with RT as a dependent variable. As expected, the analyses revealed: a main effect for range, as RTs in the subitizing range were faster than in the counting range, *F*(1, 32) = 103.24, *p* < .01, *η_p_^2^* = .77; a main effect for alertness, as RTs were faster in trials after alerting cues, *F*(1, 32) = 23.38, *p* < .01, *η_p_^2^* = .43; and a main effect for group, as RTs for controls were faster than for DD participants, *F*(1, 32) = 5.83, *p* = .02, *η_p_^2^* = .16. The main effects replicated previous findings in the literature, which ensured that our manipulations (enumeration and alerting cue) were carried out correctly. The interaction between alertness and group was significant, *F*(1, 32) = 7.36, *p* = .01, *η_p_^2^* = .19. A planned comparison of the effect of alertness separately for each group revealed, as expected, that there was a general benefit in RTs when an alerting cue preceded the target only in the DD group, namely, the DD group presented a larger alerting effect: controls – *F*(1, 32) = 2.19, *p* = .15, *η_p_^2^* = .07; DD – *F*(1, 32) = 29.38, *p* < .01, *η_p_^2^* = .51. (Note, the larger alerting effect in the DD group was because of the alerting effect in the counting range. See the relevant analysis under the triple interaction below). The interaction between range and group was found to be significant, *F*(1, 32) = 5.79, *p* = .02, *η_p_^2^* = .16. A planned comparison of the effect of group separately for each range revealed that there was a significant difference between groups only for the counting range: subitizing – *F*(1, 32) = 2.36, *p* = .13, *η_p_^2^* = .07; counting – *F*(1, 32) = 6, *p* = .02, *η_p_^2^* = .16. The interaction between alertness and range was not significant, *F*(1, 32) < 1 (but see further results in the *subitizing range analysis* below). The triple interaction between range, alertness and group was not found to be significant, *F*(1, 32) = 1.29, *p* = .26, *η_p_^2^* = .04. Because of the theoretical importance of the relationship between group, range and alertness, we examined whether alerting had a different effect between the groups for each range separately. In the subitizing range, there was no significant difference between the groups, *F*(1, 32) = 1.86, *p* = .18, *η_p_^2^* = .06, whereas in the counting range there was, *F*(1, 32) = 5.03, *p* = .03, *η_p_^2^* = .13. Further comparison between groups in the counting range revealed that alerting modulated performance only in the DD group: controls – *F*(1, 32) < 1; DD – *F*(1, 32) = 10.64, *p* < .01, *η_p_^2^* = .26 (see Figure 2). To further address the null effect for alerting in the counting range for controls, and not for the DD group, we conducted a Bayesian paired samples t-test analysis. The analysis revealed that H0 (no alertness effect in the control groups in the counting range) is 3.9 times more probable than H1 (alertness effect in the control group in the counting range).

### Subitizing range analysis

Our main goal was to examine whether an alerting cue would increase the subitizing range in those with DD. To do so, we conducted three analyses. The first analysis explored whether DD participants and controls differed in their response to the quantity of 4. The second analysis explored whether the groups differed in their slope trend. Last, we conduct a Bayesian analysis to look for a model that would predict performance of those with DD in the best way possible.

**Figure 3:**
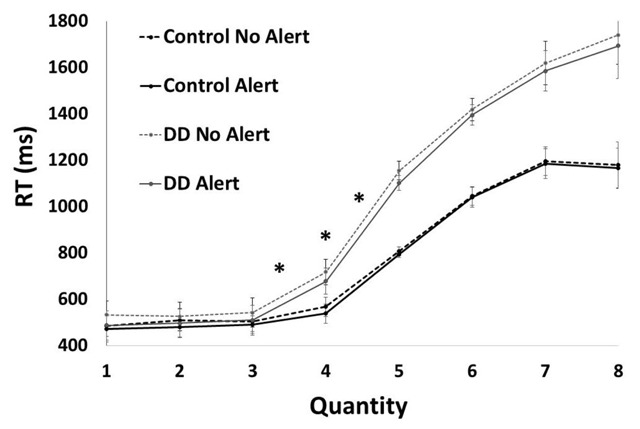
RT as a function of quantity for each group (control vs. DD) with and without alert. Asterisk (*) marks a significant difference. The subitizing range for the DD group was 3 vs. 4 for the control group. The RT slope was steeper for the DD group between quantities 3–4 and 4–5 vs. the control group.

A 2 (alertness) × 4 (quantities in the subitizing range) × 2 (groups) ANOVA was carried out, with RT as a dependent variable. The analysis revealed a significant interaction between quantity and group, *F*(3, 96) = 10.42, *p* < .01, *η_p_^2^* = .25. A planned comparison of the effect of group separately for each quantity revealed that there was a significant difference between groups only for the quantity 4 (quantities 1–3: *F*(1, 32) < 1; quantity 4: *F*(1, 32) = 10.21, *p* < .01, *η_p_^2^* = .25, See Figure 3). Moreover, in the control group the difference between RTs for quantity 3 and quantity 4 was marginally significant, *F*(1, 32) = 2.6, *p* = .07, *η_p_^2^* = .16), and in the DD group it was found to be significant, *F*(1, 32) = 70.52, *p* < .01, *η_p_^2^* = .69. The triple interaction between alertness, quantity and group was not found to be significant, *F*(3, 96) < 1.

For the second analysis, we calculated the slope between each two subsequent quantities for each participant, separately for conditions with and without alerting. A 2 (alertness) × 7 (slopes: 7 differences between 8 subsequent quantities) × 2 (groups) ANOVA was carried out, with RT-difference (slope) as a dependent variable. The analysis revealed a significant interaction between slope and group, *F*(6, 186) = 2.1, *p* = .05, *η_p_^2^* = .06. A planned comparison of the effect of group, separately for each slope, revealed differences between groups for the slopes of 3–4 and 4–5 (i.e., *F*(1, 32) = 15.6, *p* < .001, *η_p_^2^* = .33, and *F*(1, 32) = 7.9, *p* < .001, *η_p_^2^* = .2, respectively). All others planned comparison were not significant, *F*(1, 32) < 1. The analysis revealed no significant triple interaction between alertness, slope, and group, *F* < 1. A planned comparison of the effect of group separately for each slope and alerting condition revealed again differences between groups in the slopes of the quantities 3 and 4 (with alerting: *F*(1, 32) = 15.49, *p* < .001, *η_p_^2^* = .33; without alerting: *F*(1, 32) = 11.59, *p* = .001, *η_p_^2^* = .27). As can be seen in Figure 3, in the control group the trend of the slope changed between the quantities of 4 and 5, and in the DD group, the trend of the slope changed between the quantities of 3 and 4 (see Figure 3). This result is in line with the accuracy analysis. Additionally, a difference was found in the slope between quantities 4 and 5, as the slope was steeper in the DD group (with alerting: *F*(1, 32) = 6.82, *p* < .01, *η_p_^2^* = .18; without alerting: *F*(1, 32) = 7.8, *p* < .01, *η_p_^2^* = .2). All others planned comparison were not significant (with alerting, for 1–2, 2–3, 5–6, 6–7: *F* < 1; for 7–8: *F*(1, 32) = 1.7, *p* = .19; without alerting, for 1–2: *F*(1, 32) = 1.3, *p* = .27; for 2–3: *F*(1, 32) = 2.7, *p* = .11; for 5–6, 6–7: *F* < 1; for 7–8: *F*(1, 32) = 1.7, *p* = .2).

Last, we conducted a Bayesian repeated measures ANOVA with the quantities 3 and 4, with and without alerting, in the DD group. The analysis revealed that a model that includes only the main effects (alerting and quantity) explained the results better than did a model that included the mains effect and the interaction between them. Namely, the probability that H_0_ (null effect, alerting cue did not change the subitizing range) was 2.82 more likely than the probability that H_1_ was correct. Note that the Bayes factor value is considered to be close to substantial (Wetzels et al., 2011), and is in line with the other analyses.

To summarize, our main finding is that controls and those with DD differ in their subitizing range (4 vs. 3, respectively), and that an alerting cue did not change the subitizing range of the DD group.

## Discussion

The present study was aimed at examining the involvement of attention in enumeration processes in adults with developmental dyscalculia. Let us summarize the main results: 1) For controls, alerting increased the accuracy rate and facilitated enumeration of quantities in the subitizing range, but not in the counting range. 2) DD participants presented a larger alerting effect than controls did, and alerting effect appeared for both ranges. 3) DD participants presented deficiencies in the subitizing range. Their accuracies were lower than controls for quantities 3, 4, and 5, and the change from a low to a relatively high slope appeared between quantities 3 and 4 rather than between 4 and 5. 4) For DD participants, an alerting cue facilitated general RTs but did not change their accuracy rates or their smaller than normal subitizing range.

As for controls, the results of the current study replicated the results of the effect of alerting on enumeration reported by Gliksman et al. (2016). Namely, alerting cues affected RTs for subitizing only. There is strong evidence suggesting that alerting induces a global processing bias in a normative population (Weinbach & Henik, 2011), in a population with developmental disorders (Kalanthroff, Naparstek, & Henik, 2013; Tanzer, Weinbach, Mardo, Henik, & Avidan, 2016), and acquired disorders (Van Vleet et al., 2011). It was suggested that subitizing involves pattern recognition (Ashkenazi, Mark-Zigdon, et al., 2013; Mandler & Shebo, 1982) because quantities in this range can be identified as familiar global configurations. Therefore, the current results support the suggestion that alerting could facilitate the subitizing process by enhancing global processing.

In the current study, DD participants presented atypical attention abilities. Previous research reported that participants with DD presented higher percentages of omission and commission errors in the CPT, in addition to longer RTs and larger variability in their responses. Their performance was correlated to lower arithmetic achievement scores (Lindsay, Tomazic, Levine, & Accardo, 2001). Specifically, regarding the alerting network of attention (Petersen & Posner, 2012; Posner & Petersen, 1990), Ashkenazi and Henik (2010) tested the performance of those with DD in the ANT-I (Callejas et al., 2004) and reported a deficit in the network of alerting attention. Our finding of a larger alerting effect in the DD group is in line with their report. It should be noted that our sample of DD participants were recruited according to criteria of pure DD. Namely, there were no indications of attentional deficit according to both questionnaires and computerized tests (Ben-Simon et al., 2008). However, the DD group’s results in the current study support former reports on a comorbidity relation between arithmetic and attentional deficits (Ashkenazi & Henik, 2010).

Our results can contribute to the discussion of domain-general and domain-specific effects in numerical operations (e.g., enumeration) and the involvement of those processes in DD. On one hand, participants with DD presented atypical attention abilities, supporting the involvement of domain-general processes. On the other hand, DD participants presented a smaller than normal subitizing range, and the attentional manipulation facilitated general responding but did not change the DD group’s subitizing range or accuracy rates. The latter supports involvement of domain-specific processes in the disorder. Note that a similar conclusion was suggested by Ashkenazi and Henik (2012). The authors trained DD participants and controls in video games, which were designed in order to improve attention abilities. Before training, the DD group presented a larger alerting effect. After 10 days of training, the DD group presented an alerting effect similar to controls and showed a reduced RT. However, the subitizing range of the DD group did not change. While Ashkenazi and Henik trained participants in overall attentional abilities, here we specifically manipulated the alerting system. Yet, the same results were demonstrated in both studies.

Importantly, we do not suggest that enumeration or DD should be considered solely as a domain-specific or domain-general deficit. Instead, we would like to emphasize that enumeration is a complex operation, which is based on both domain-specific and domain-general mechanisms. This suggestion is in line with recent findings reporting that a general mechanism of visuo-spatial working memory is related to enumeration in the subitizing range (Piazza et al., 2011), and with the reporting that both domain-general and domain-specific abilities uniquely contributed to complex arithmetic tasks (Ashkenazi, Golan, & Silverman, 2014), and fits with recent theories suggesting that DD is a heterogeneous phenomenon that may have different origins, related to numerical processing and to other mechanisms (Kaufmann et al., 2013; Rubinsten & Henik, 2009).

Interestingly, the rTPJ (right tempo-parietal junction) was found to be involved in all mentioned cognitive mechanisms. Regarding alerting, it was reported that the rTPJ was involved in effects of visual and auditory alerting cues (Robertson, Mattingley, Rorden, & Driver, 1998; Thiel & Fink, 2007). Regarding global processing, the right hemisphere was found to be related (Van Kleeck, 1989), and studies of patients with focal injury in the rTPJ have demonstrated a deficit in global processing (Lamb, Robertson, & Knight, 1989, 1990). Regarding subitizing, the rTPJ was found to be activated when participants performed an enumeration task in the subitizing range and was inhibited in the estimation range (Ansari, Lyons, van Eimeren, & Xu, 2007). Moreover, it was reported that those with DD had reduced gray matter in the rTPJ (Rykhlevskaia, Uddin, Kondos, & Menon, 2009). Taken together, a developmental deficit in the rTPJ might explain the poor performance of those with DD in both alerting and subitizing mechanisms. Future studies need to examine whether manipulation of activity in the rTPJ in DD participants will increase their smaller than normal subitizing range.

A limitation that should be address in our results is the null effect: an alerting cue did not change the subitizing range of the DD group. The null effect could result from an insufficient statistical power, or because the introduced manipulation (the alerting cue in our study) did not create a difference. Bayesian analysis can shed light on this and help determine what the probability is that H_0_ (alerting cue did not change the subitizing range of the DD participants), and not H_1_ (alerting cue changed the subitizing range of the DD participants), better described the result. In our study, this analysis suggested that H_0_ is about 3 times more likely than H_1_. Other analyses supported this result too. Nevertheless, further research should examine whether the small subitizing range of those with DD can be improved.

## Conclusions

The present study demonstrated the involvement of attention in enumeration processes in adults with and without developmental dyscalculia. Participants with DD presented a domain-specific deficit in the enumeration process in addition to a domain-general deficit in attentional processes. It seems that numerical operations and developmental disorders involve both aspects.

## Additional File

The additional file for this article can be found as follows:

10.5334/joc.55.s1 Supplemental Materials. MATAL diagnosis – developmental dyscalculia (DD).

## Data Availability

Data of the experiment will appear in an open preservation repository. Data of the MATAL diagnosis of the participants with DD will be publish as a group and not per participant due to ethical reasons.
