# Supplementary material for: Enumeration and Alertness in Developmental Dyscalculia
Source: J Cogn. 2019 Feb 4;2(1):5. doi: 10.5334/joc.55 (PMC6634361; doi:10.5334/joc.55)
Supplement: Supplemental Materials. — MATAL diagnosis – developmental dyscalculia (DD). [file joc-2-1-55-s1.pdf]

Enumeration and Alertness in Developmental Dyscalculia

Supplemental Materials

MATAL diagnosis – developmental dyscalculia (DD)

Table 1: Results of subtests in the MATAL diagnosis of DD participants.

	Subtest	DD score
Arithmetic		
	Retrieval of arithmetic facts RT	-4.3 (1)
	Retrieval of arithmetic facts ACC	-4.1 (2)
	Procedural knowledge RT	-2.3 (1)
	Procedural knowledge ACC	-4.3 (1)
	Number estimation RT	-2.3 (1)
	Number estimation ACC	-2.7 (1)
Reading		
	Vocal text reading RT	.3 (1)
	Vocal text reading ACC	.1 (.8)
	Phonemic deletion RT	0 (.7)
	Phonemic deletion ACC	0 (.6)
	Phonemic counting RT	.1 (1)
	Phonemic counting ACC	.2 (.6)
	Non-word reading RT	-.3 (.7)
	Non-word reading ACC	-.4 (.8)
	Rapid automatic naming (objects)	-.2 (1)
Attention		
	CPT omitting	.1 (.3)

CPT commission	.2 (.9)
CPT RT	.1 (1)
CPT *SD* of RT	.2 (1)
Attention abilities (ANT) RT	.1 (.7)
Attention abilities (ANT) ACC	-.7 (.2)

Note. Scores are reported as Z-scores (mean = 0, *SD* = 1); standard deviation in parentheses; CPT = Continuous Performance Test; RT = reaction time; ACC = accuracy; ANT = Attention Network Test.

Matching DD participants to controls - Arithmetic Battery and Additional Tests

Part 1: Number comprehension and production

a. *Comparing digits.* Comparison of eight pairs of numbers: two pairs of 3-digit numbers, three pairs of 4-digit numbers and three pairs of 5-digit numbers. The participants had to mark a circle on the largest number.

b. *Counting forward and backward.* The participant had to count forward seven times (e.g., count from 793-802) and backward four times (e.g., count from 813-798). The initial number was a 2-, 3- or 4-digit number.

c. *Serial order*. Participants were presented with 14 arithmetical series and had to fill the three numbers in the sequence, from largest to smallest and from smallest to largest.

d. *Comparing fractions.* The participants were presented with eight pairs of decimals and had to circle the largest number.

e. *Verbal problems*. Eight verbal problems were presented and participants were asked to: 1) Circle the correct operation for the given problem (addition, subtraction, multiplication or division). 2) Write down the representative equation. 3) Solve the equation. For example: In a building, there are 31 floors. The height of each floor is 4 meters. What is the height of the building? Answer: step 1 - the correct operation is multiplication; step 2 - the correct equation is: 31 × 4 = ; and step 3 - the correct answer is: 124.

f. *Procedural knowledge*. Participants were presented with 8 horizontal equations and were asked to copy them to vertical equations. Four equations were composed of real numbers and four equations were composed of decimals. The real number equations included two, three or four numbers of 1-, 2-, 3- or 4-digit numbers. For example 21,306+28+2+236= for the real numbers and 3.27 + 0.365 = for the decimal equations.

Part 2 - Calculation

a. *Simple pure operations*. Single-digit operations were administered (10 additions, 10 subtractions, 10 divisions and 10 multiplications). Each kind of operation appeared separately.
b. *Simple mixed operations*. Single-digit operations were administered (five additions, five subtractions, five divisions and five multiplications), mixed in one block in a random order. The equations had the same level of difficulty as in the pure block.
c. *Decimal*. There were four complex addition and four complex subtraction exercises requiring knowledge of decimals. The exercises were presented vertically. The numbers contained two digits after the decimal point.
d. *Estimation*. The experimenter asked participants to estimate the results of an equation (four additions, four subtractions, four divisions and four multiplications), or to decide whether the result would be bigger or smaller than a specific number. For example, estimate the result of 589+426.
e. *Comparing equations.* Participants were asked to decide which equation would result larger number. Participants were instructed to decide according to estimation and not accurate calculation. There were eight comparisons (two additions, two subtractions, two divisions and two multiplications). For example, 71-38 or 73-28.
f. *Vertical operation.* Participants had to solve complex written arithmetic problems (addition, subtraction, multiplication, and division). The first eight exercises were addition and subtraction, and the remaining eight were multiplication and division. Exercises included problems with and without borrowing using each operation.

Additional tests – Controls and DD participants

Table 2: IQ, Attention and reading tests.

	**Controls**	**DD**	***t***	***p***
Raven (correct answers, out of 60)	47.9 (4.9)	47.4 (6.5)	.22	.82
CPT (RT)	.11 (11)	.12 (19)	-1.1	.28
CPT (ACC)	94 (3.8)	94 (3.6)	-.9	.36
Reading test (words per minute)	127 (13)	126 (17)	.26	.8
Reading test (ACC)	100 (0.4)	99 (0.7)	1.2	.23
Non-Word (RT)	36 (13)	41 (16)	.42	.68
Non-Word (ACC)	95 (13)	85 (16)	1.78	.09

Note. Standard deviations in parentheses; CPT = Continuous Performance Test; RT = reaction time; ACC = accuracy.

Table 3: Arithmetic battery Part A: Number comprehension and production.

	**Controls**	**DD**	***t***	***p***
Comparing digits (RT)	11 (2.7)	24 (8.3)	-5.5	<.001
Comparing digits (ACC)	99 (3.2)	99 (3.2)	.05	.96
Counting forward (RT)	52 (10)	83 (28)	-3.9	<.001
Counting forward (ACC)	88 (9)	81 (11)	1.77	.08
Counting backwards (RT)	49 (12)	76 (19)	-4.54	<.001
Counting backwards (ACC)	92 (12)	75 (17)	3.09	.004
Serial order (RT)	85 (34)	318 (5)	-25.5	<.001
Serial order (ACC)	97 (5)	91 (9)	2.16	.039
Comparing fractions (RT)	12 (3)	40 (18)	-5.89	<.001
Comparing fractions (ACC)	98 (10)	80 (31)	2.02	.05
Verbal problems (RT)	201 (78)	392 (146)	-4.4	<.001
Verbal problems (ACC)	85 (8)	54 (19)	5.73	<.001
Procedural knowledge (RT)	159 (56)	261 (145)	-2.58	.016
Procedural knowledge (ACC)	84 (13)	47 (22)	5.5	<.001

Note. Standard deviations in parentheses; RT = reaction time; ACC = accuracy.

Table 4: Arithmetic battery Part B: Calculation.

	**Control**	**DD**	***t***	***p***
Simple Operations				
Addition (RT)	15 (6)	34 (13)	-5.2	<.001
Addition (ACC)	99 (3)	94 (13)	1.15	.27
Subtraction (RT)	11 (5)	33 (14)	-5.6	<.001
Subtraction (ACC)	99 (2)	98 (4)	1.06	.3
Multiplication (RT)	16 (7)	102 (79)	-4.22	.001
Multiplication (ACC)	98 (5)	77 (21)	3.79	.002
Division (RT)	18 (11)	99 (92)	-3.38	.002
Division (ACC)	96 (5)	67 (28)	3.9	.001
Mixed operations (RT)	58 (31)	212 (149)	-3.95	<.001
Mixed operations (ACC)	92 (10)	71 (19)	3.8	.001
Decimals (RT)	72 (37)	123 (53)	-3.07	.005
Decimals (ACC)	88 (13)	58 (33)	3.28	.004
Estimation (ACC)	77 (13)	45 (19)	5.35	<.001
Comparing equations (RT)	42 (13)	104 (20)	-9.76	<.001
Comparing equations (ACC)	90 (10)	82 (17)	1.47	.16
Vertical operation (RT)	56 (18)	141(53)	-5.89	<.001
Vertical operation (ACC)	100 (0)	91 (18)	1.78	.1

Note. Standard deviations in parentheses; RT = reaction time; ACC = accuracy.
